# Supplementary material for: Identification of photoperiod-induced specific miRNAs in the adrenal glands of Sunite sheep (Ovis aries)
Source: Front Vet Sci. 2022 Jul 22;9:888207. doi: 10.3389/fvets.2022.888207 (PMC9354845; doi:10.3389/fvets.2022.888207)
Supplement: Supplementary file 1 [file Data_Sheet_1.ZIP › Supplementary file/miRDeep2 file.zip!/miRDeep2 file/Know_Structure/LP42A1/oar-mir-125b.pdf]

miRBase precursor	: oar-mir-125b
Total read count	: 212486
oar-miR-125b read count	: 211892
remaining reads	: 594

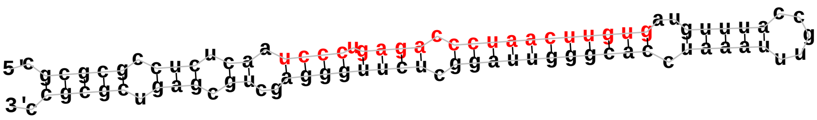

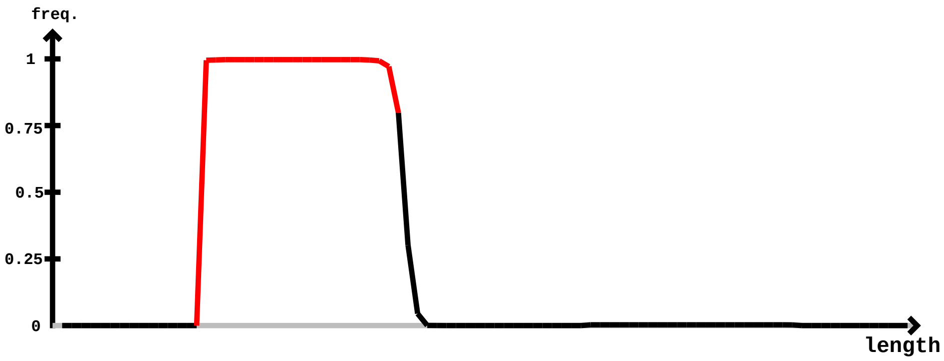

oar-miR-125b

```
5'- cgcgcgccucucaaucccugagacccuaacuugugauguuuaccguuuaaauccacggguuaggcucuugggagcugcgagucgcgcc   -3'    exp
    .(((((.(((.((.((((.((((.(((((((((((..(((((.....))))).))))))))))).))))))))..)).))).))))).    reads      mm          sample
    ............aaucccugagacccuaacuugug.....................................................    1          0           seq
    ............aaucccugagacccuaacuuguga....................................................    1          0           seq
    .............aucccugagacccuaacuug.......................................................    1          0           seq
    .............aucccugagacccuaacuugu......................................................    1          0           seq
    .............aucccugagacccuaacuugug.....................................................    5          0           seq
    ..............ucccugagacccuaacu.........................................................    269        0           seq
    ..............ucccugagacccuGacu.........................................................    1          1           seq
    ..............ucccugagacccuaacC.........................................................    38         1           seq
    ..............Acccugagacccuaacu.........................................................    1          1           seq
    ..............uUccugagacccuaacu.........................................................    1          1           seq
    ..............ucccugagacccuaCcu.........................................................    2          1           seq
    ..............ucccugagGcccuaacu.........................................................    2          1           seq
    ..............ucccugagaUccuaacu.........................................................    3          1           seq
    ..............ucccugagacccuaacCu........................................................    7          1           seq
    ..............ucccugagacccuaacuu........................................................    640        0           seq
    ..............ucccugagacccuaacuug.......................................................    4521       0           seq
    ..............ucccugagacccuaacuugu......................................................    36782      0           seq
    ..............ucccugagacccuaacuugug.....................................................    105290     0           seq
    ..............ucccugagacccuaacuuguga....................................................    54375      0           seq
    ..............ucccugagacccuaacuugugau...................................................    6388       0           seq
    ..............ucccugagacccuaacuugugaG...................................................    2984       1           seq
    ..............ucccugagacccuaacuugugaug..................................................    30         0           seq
    ..............ucccugagacccuaacuugugaGg..................................................    25         1           seq
    ..............ucccugagacccuaacuugugaGgu.................................................    2          1           seq
    ..............ucccugagacccuaacuugugaugu.................................................    41         0           seq
    ..............ucccugagacccuaacuugugauguu................................................    1          0           seq
    ...............cccugagacccuaacuugu......................................................    6          0           seq
    ...............cccugagacccuaacuugug.....................................................    22         0           seq
    ...............cccugagacccuaacuuguga....................................................    198        0           seq
    ...............cccugagacccuaacuugugau...................................................    9          0           seq
    ...............cccugagacccuaacuugugaG...................................................    6          1           seq
    ...............cccugagacccuaacuugugaGg..................................................    1          1           seq
    ...............cccugagacccuaacuugugaug..................................................    1          0           seq
    ................ccugagacccuaacuug.......................................................    6          0           seq
    ................ccugagacccuaacAugu......................................................    1          1           seq
```

oar-miR-125b

```
cgcgcgccucucaaucccugagacccuaacuugugauguuuaccguuuaaauccacggguuaggcucuugggagcugcgagucgcgcc
................ccugagacccuaacuugu.....................................................    37     0     seq
................ccugagacccuaacuugug....................................................    117    0     seq
................ccugagacccuaacuuguga...................................................    61     0     seq
................ccugagacccuaacuugugaG..................................................    3      1     seq
................ccugagacccuaacuugugau..................................................    5      0     seq
.................cugagacccuaacuugu.....................................................    1      0     seq
.................cugagacccuaacuugug....................................................    1      0     seq
.................cugagacccuaacuuguga...................................................    4      0     seq
.................cugagacccuaacuugugau..................................................    1      0     seq
.....................................................cacggguuaggcucuugggagc.............    1      0     seq
......................................................acggguuaggcucuuggg................    1      0     seq
......................................................acggguuaggcucuuggga...............    1      0     seq
......................................................acggguuaggcucuugggag..............    4      0     seq
......................................................acggguuaggcucuugggagc.............    55     0     seq
......................................................acggguuaggcucuugggagcu............    524    0     seq
......................................................acggguuaggcucuugggagcug...........    8      0     seq
```
